# Pan-cancer transcriptional atlas of minimal residual disease links DUSP1 to chemotherapy persistence

**DOI:** 10.1186/s40164-024-00509-3

**Published:** 2024-04-16

**Authors:** Yuanhui Liu, Bi Peng, Ziqi Chen, Yimin Shen, Jingmin Zhang, Xianglin Yuan

**Affiliations:** 1 Department of Oncology, Tongji Hospital, Tongji Medical College, Huazhong University of Science and Technology, Wuhan, Hubei China; 2 Cancer Center, Tongji Hospital, Tongji Medical College, Huazhong University of Science and Technology, Wuhan, Hubei China; 3 Laboratory for Bioinformatics, Fondation Jean Dausset - CEPH, Paris, France; 4 Hubei Hongshan Laboratory, College of Biomedicine and Health, Huazhong Agricultural University, Wuhan, Hubei China

**Keywords:** Pan-cancer, Chemotherapy, Minimal residual disease, DUSP1

## Abstract

Chemotherapy is a commonly effective treatment for most types of cancer. However, many patients experience a relapse due to minimal residual disease (MRD) after chemotherapy. Previous studies have analyzed the changes induced by chemotherapy for specific types of cancer, but our study is the first to comprehensively analyze MRD across various types of cancer. We included both bulk and single-cell RNA sequencing datasets. We compared the expression of the entire genome and calculated scores for canonical pathway signatures and immune infiltrates before and after chemotherapy across different types of cancer. Our findings revealed that DUSP1 was the most significantly and widely enriched gene in pan-cancer MRD. DUSP1 was found to be essential for MRD formation and played a role in T cell-fibroblast communications and the cytotoxic function of CD4 ^+^ T cells. Overall, our analysis provides a comprehensive understanding of the changes caused by chemotherapy and identifies potential targets for preventing and eliminating MRD, which could lead to long-term survival benefits for patients.

**Supplementary Information:**

The online version contains supplementary material available at 10.1186/s40164-024-00509-3.

## To the editor,

Chemotherapy is the conventional and widely accepted treatment for most types of cancer. Some patients can achieve complete regression with chemotherapy. However, one of the most important causes of relapse and treatment failure is minimal residual disease (MRD) [1]. Previous efforts to study MRD have been limited to specific single cancer types, such as breast cancer (BRCA), rectal cancer (READ) and ovarian cancer (OV) [2–4]. There has been a lack of analysis of MRD across multiple types of cancer. Recent advancements in genomic technologies and large-scale data analysis [5, 6] make it possible to conduct a comprehensive analysis of pan-cancer MRD. Our goal is to investigate MRD in various cancer types in order to gain a more complete understanding of its role in disease progression and response to treatment.

**Fig. 1:**
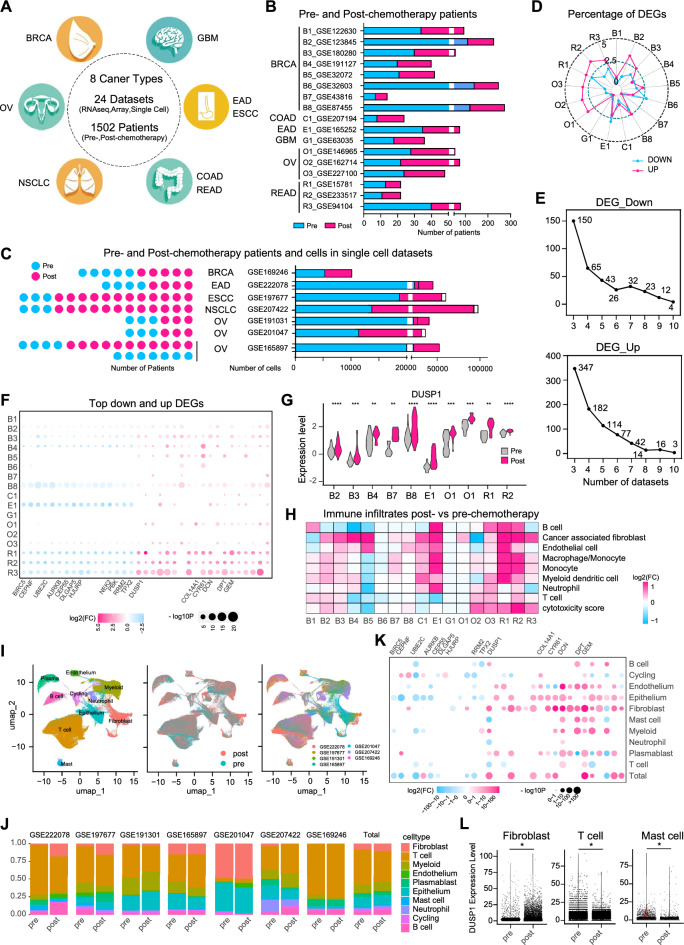
Pan-cancer pre- and post-chemotherapy multi-omics analysis identified DUSP1 as a target enriched in minimal residual disease. **A** Schematic depicting the study design. We utilized 24 published datasets, consisting of 17 bulk RNAseq and array datasets, as well as 7 single cell RNA seq datasets. These datasets were obtained from 8 different cancer types and included a total of 1502 patients. BRCA: breast cancer, OV: ovarian cancer, NSCLC: non-small cell lung cancer, GBM: gliomablastoma, EAD: esophageal adenocarcinoma, ESCC: esophageal squamous cell carcinoma, COAD: colon adenocarcinoma, READ: rectal adenocarcinoma. **B** Bar graphs displaying the number of patients before and after chemotherapy in various bulk RNAseq and array datasets. **C** Bubble and bar graphs showing number of pre- and post-chemotherapy patients and cells across various single cell dataset. **D** Radar plot showing the percentage of genes that are significantly down-regulated (p < 0.05, log2(FC) < -1) and up-regulated (p < 0.05, log2(FC)- > 1) in patients who received chemotherapy compared to those who did not. The data is collected from 17 datasets representing 6 different types of cancer. BRCA: B1-B8, B1,GSE122630; B2, GSE123845; B3, GSE180280; B4, GSE191127; B5, GSE32072; B6, GSE32603; B7, GSE43816; B8, GSE87455; COAD: C1, GSE207194; EAD: E1, GSE165252; GBM: G1, GSE63035; OV: O1-O3, O1, GSE146965; O2, GSE16274; O3, GSE227100; READ: R1-R3, R1, GSE15781; R2, GSE233517; R3, GSE94104. **E** Genes found to be recurrently significantly differentially expressed between post- and pre-chemotherapy patients in multiple datasets. **F** Bubble plot showing the log2(FC) and -log10p of 35 most widely differentially expressed genes (DEGs) across 17 datasets. The marked down and up DEGs are related to cell cycle and ECM pathway respectively. **G** Violin plot comparing the expression changes of DUSP1 between patients samples before and after chemotherapy. Two sided Welch’s t-test was applied to calculate p values: (*p < 0.05, **p < 0.01, ***p < 0.001, ****p < 0.0001). **H** Heat map shows the log2(FC) of immune infiltrates (calculated by MCPcounter method) between post- and pre-treatment patients across 17 datasets. Color represents log2(FC). **I** UMAP embedding overlaid with unsupervised cluster cell type annotations (left), treatment annotations (medium) and dataset annotations (right) of totally 323,664 cells integrating seven datasets. **J** The cell type composition in pre- and post-chemotherapy group of samples across various datasets and in the integrity. **K** Bubble plot showing the log2(FC) and -log10p of 35 most widely differentially expressed genes (DEGs) across 10 cell types and the integrity. **L** Comparison of DUSP1 expression between pre- and post-chemotherapy patient samples across fibroblast, T cell and mast cell. *p < 0.05

To clarify the changes induced by chemotherapy and develop strategies to tackle with MRD, we collected bulk RNA-seq, array data as well as single cell RNA-seq from 24 datasets and 8 cancer types involving 1502 individuals with samples taken before and after chemotherapy (Fig. 1A). The 17 bulk RNA-seq and array datasets included 14–275 patients (Fig. 1B, Additional file 1: Table S1). A total of 323,664 cells from 65 patients across 7 single cell RNA-seq datasets were analyzed (Fig. 1C). The differential expression analysis was performed to characterize the MRD induced by chemotherapy. The percentage of significant differential expressed genes (DEGs) out of genome was calculated (Fig. 1D). Among the down-regulated genes in MRD, 16 genes were common in more than nine datasets, while 19 genes were up-regulated in at least nine datasets (Fig. 1E, F, Additional file 2: Table S2). Many of these down-regulated genes are associated with the regulation of the cell cycle, while a few of the up-regulated ones are associated with the extracellular matrix (ECM) pathway (Fig. 1F). Notably, DUSP1 emerged as the most up-regulated gene in ten datasets (Fig. 1G). Signature scores of canonical pathways were calculated, revealing that cell cycle-related pathways were the most depleted in MRD (Additional file 5: Fig. S1A, Additional file 3: Table S3).

We then analyzed the dynamic changes in tumor-infiltrating lymphocytes using the MCPcounter algorithm [7]. We found that cancer-associated fibroblasts (CAFs) and myeloid cells were more abundant in minimal residual disease (MRD), while T cells were down-regulated in some tumors after chemotherapy (Fig. 1H and Additional file 4: Table S4). We also examined the expression of immune regulators and immunogenic cell death following chemotherapy. The results showed a general increase in immune inhibitory and stimulatory regulators in the MRD of READ, along with a decrease in the expression of HMGB1 and HSP90AA1 (Additional file 5: Fig. S1B, C).

We then aimed to compare the relationship between gene expression and immune infiltrates in patients before and after chemotherapy, with their response to chemotherapy and clinical outcomes. The distribution of z scores was similar in the pre-chemotherapy (control) and post-chemotherapy (MRD) groups (Additional file 5: Fig. S2A). The number of genes that can predict outcomes in both the pre- and post-chemotherapy samples is small (Additional file 5: Fig. S2B–E). We observed a positive correlation between the changes in gene expression after chemotherapy and their ability to predict overall survival (Additional file 5: Fig. S2F). The prediction direction for immune infiltrates is reversed when comparing pre- and post-chemotherapy samples (Additional file 5: Fig. S2G). In general, the features in the post-chemotherapy group do not outperform those in the pre-chemotherapy group.

To gain a better understanding of minimal residual disease (MRD) at the single-cell resolution and in the microenvironment, we analyzed seven single-cell RNA sequencing datasets totaling 323,664 cells (Fig. 1I). We observed an increase in CAFs and a decrease in T cells in MRD following chemotherapy (Fig. 1J). The 35 genes that showed the most significant change following chemotherapy in bulk RNA sequencing were mostly confirmed in the single-cell datasets (Fig. 1K). We then focused on the DUSP1, that is previously reported to be related to drug sensitivity [8, 9] and identified here as most up-regulated gene upon chemotherapy from bulk RNA-seq analysis. The expression of DUSP1 was found to increase in CAFs and decrease in T cells and mast cells (Fig. 1L, Additional file 6: Table S5).

**Fig. 2:**
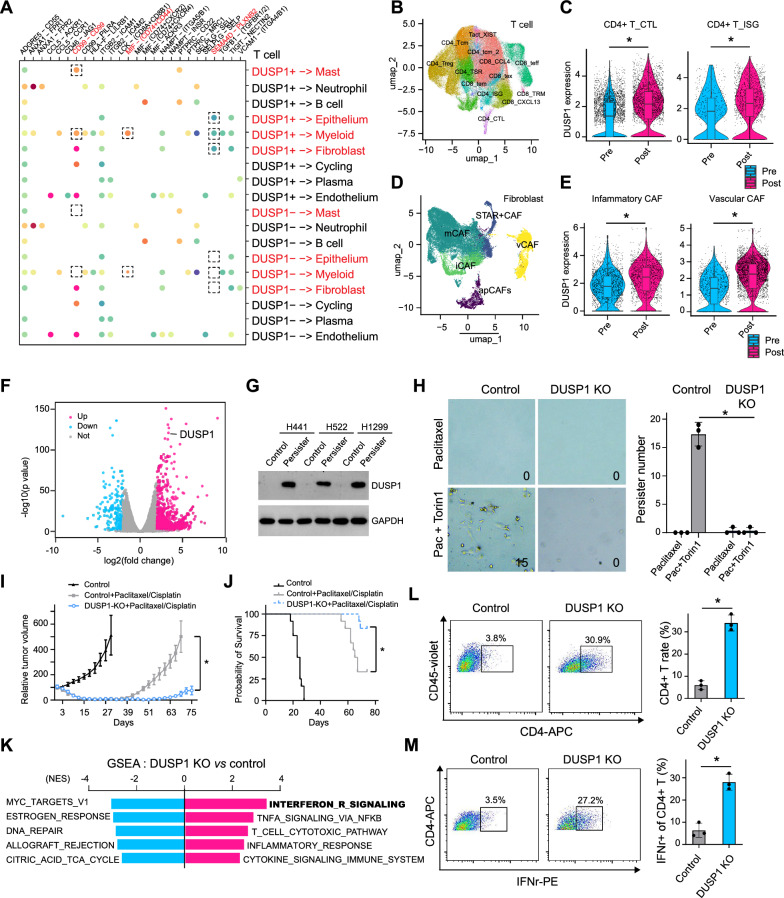
DUSP1 is indispensable for minimal residual disease and CD4^+^ T cell cytotoxity. **A** Significant ligand-receptor pairs of cell communication between T cells, which were grouped as DUSP1^+^ and DUSP1^−^, with other cell types. **B** UMAP embedding overlaid with unsupervised cluster cell type annotations of T cells integrating seven datasets. **C** DUSP1 expression in pre- and post-chemotherapy patient sample in CD4^+^ T_CTL cell (left) and CD4^+^ T_TSG cell (right). *p < 0.05. **D** UMAP embedding overlaid with unsupervised cluster cell type annotations of fibroblast cells integrating seven datasets. **E** DUSP1 expression in pre- and post-chemotherapy patient sample in inflammatory CAF cell (left) and vascular CAF cell (right). *p < 0.05. **F** Volcano plot showing the gene profile change between persister and control cells of RNA seq results. The red dots represent the genes that are significantly up-regulated in persister cells, and the blue dots represent the genes that are significantly down-regulated in persister cells. **G** Western blot showing DUSP1 expression in persister cells of three different cell line. **H** Microscopic images of human cancer cells treated with Torin1 plus chemotherapeutic agents (10 nM paclitaxel). The images are representative of three biological replicates. The average cell count per image is indicated in the lower right corner. Scale bar, 100 μm. The column blot shows the number of persister. *p < 0.05. **I** Tumor growth of mouse subcutaneous xenograft model using control or DUSP1 KO cells treated or not with chemotherapeutics. The minimal residual disease and relapase time line is shown across groups. *p < 0.05. **J** Survival of mice bearing wild type and DUSP1 knockout tumors with or without chemotherapeutics treatment for 4 weeks. *p < 0.05. **K** GSEA results using the canonical pathway gene sets in DUSP1 KO vs wild type pair. Normalized enrichment scores (NES). **L**, **M**. Flow cytometry was used to analyze the proportion and cytotoxicity of CD4^+^ T cells in the DUSP1 KO and wild type cells. *p < 0.05

To investigate the mechanism by which DUSP1 influence micro-environment and minimal residual disease, we examined the communication between T cells (divided into two groups based on DUSP1 expression: DUSP1^+^ and DUSP1^−^) and other cell types. It was observed that DUSP1^+^ T cells communicated with CAFs specifically through the SEMA4D–PLXNB2 ligand-receptor group (Fig. 2A). Additionally, DUSP1^+^ T cells, but not DUSP1^−^ T cells, communicated with myeloid cells through the CD99–CD99, MIF–(CD74 + CD44), and SEMA4D–PLXNB2 ligand-receptor groups (Fig. 2A). We then focused on the T cells and CAFs, and integrated a total of 163,101 and 35,742 cells respectively (Fig. 2B, D). DUSP1 was found to be enriched in CD4^+^ cytotoxic T cells (CTL) and CD4^+^ T cells with an interferon response (ISG), suggesting its possible involvement in CD4^+^ T cell cytotoxic function (Fig. 2C). The enrichment of DUSP1 was also seen in inflammatory and vascular CAFs, which indicates the correlation between DUSP1 and tumor angiogenesis (Fig. 2E).

To further investigate the role of DUSP1 in drug tolerance, we utilized the drug-tolerant persister (DTP) model previously described in our study [10]. Briefly, we treated the cells with chemotherapeutic agent at LD100 combined with mTOR inhibitors to induce the DTP cells. We examined the exchange of transcriptional profiles and protein expression between DTP and control cells and found that DUSP1 was more abundant in persister cells (Fig. 2F, G). And persister cell is compromised in the DUSP1 knockout (KO) condition (Fig. 2H). In an in vivo mouse model, the lack of DUSP1 led to a reduction in minimal residual disease, resulting in a longer relapse-free period (Fig. 2I) and overall survival (Fig. 2J). Interestingly, the transcriptional profiles of DUSP1 KO cells showed significant enrichment for IFN response signaling and the T cell cytotoxicity pathway, as indicated by Gene Set Enrichment Analysis (GSEA) of 2900 canonical signaling pathways (Fig. 2K). Flow cytometry analysis further demonstrated a notable increase in the proportion of CD4^+^ T cells and their ability to secrete IFN-γ in the absence of DUSP1 (Fig. 2L, M).

Although a few studies have investigated the changes induced by chemotherapy [11, 12], this type of analysis is problematic due to the limited number of tumors analyzed and technical challenges that increase the likelihood of false discoveries. Here, we thoroughly examined the multi-omic profile of MRD and found that DUSP1 is highly enriched in MRD. We demonstrated that DUSP1 is indispensable for MRD induction and immuno-suppresive micro-environment formation, thus identifing DUSP1 as a pan-cancer target for the strategy of preventing and eliminating MRD.

### Supplementary Information

**Additional file 1: Table S1.** Clinical information of the 1440 patients across 17 datasets.

**Additional file 2: Table S2.** Illma differential expression analysis of mRNA expression.

**Additional file 3: Table S3.** Illma differential expression analysis of signature score.

**Additional file 4: Table S4.** Illma differential expression analysis of immune infiltrates.

**Additional file 5: Figure S1.** Canonical pathway signature scores, immune infiltrates and modulators changes induced by chemotherapy. A Heat map illustrating log2(FC) of 10 most widely significantly differentially expressed canonical pathway signature scores across 17 datasets. B Heat map shows the changes in expression (log2(FC)) of inhibitory and stimulatory immune modulators in patients before and after chemotherapy. C Heat map shows the changes in expression (log2(FC)) of seven immunogenic cell death (ICD) modulators between post- and pre-chemotherapy patients. **Figure S2.** Predictivity of features in pre- and post-chemotherapy samples for drug response and survival. A Distrbution of z score of whole transcriptomic genes for the prediction of overall survival (OS), recurrence free survival (RFS), recurrence (Re) and drug response (DR) in pre-chemotherapy and post-chemotherapy patients samples across nine datasets in three cancers. B Radar plot showing the percentage of significant gene (p<0.05) for the prediction of OS, RFS, Re and DR in pre-chemotherapy and post-chemotherapy patients samples across 9 datasets in three cancers. C Bar plot displaying the fraction of shared significant prognostic genes (overlap) between significant genes derived from patient samples before chemotherapy and significant genes derived from patient samples after chemotherapy. D The number of significant prognostic genes in patient samples before and after chemotherapy was compared across nine datasets in three types of cancer. Each dot represents one dataset. E Kaplan-Meier plots displaying the prognostic ability of ADH in pre-chemotherapy patient sample and in post-chemotherapy patient sample in dataset GSE146965. F Pearson correlation between the gene expression changes (log2(FC) of post- vs pre-chemotherapy) and the z score of genes in COX regression models for OS in pre-chemetherapy patient samples. G Heat map showing the z score of immune infiltrates for prediction of OS, RFS, Re and DR in pre-chemotherapy and post-chemotherapy patients samples across nine datasets in three cancers.

**Additional file 6: Table S5.** P value and logFC of differential gene expression analysis results of single cell sequencing datasets.

## Data Availability

Publically available transcriptome-level array or bulk RNA-seq gene expression datasets were retrieved from Gene Expression Omnibus (GEO) with the following accession numbers: GSE122630, GSE123845, GSE180280, GSE191127, GSE32072, GSE23603, GSE43816, GSE87455, GSE207194, GSE165252, GSE63035, GSE146965, GSE162714, GSE227100, GSE15781, GSE233517, GSE94104. The scRNA-seq datasets were retrieved from Gene Expression Omnibus (GEO) with the following accession numbers: GSE169246, GSE191301, GSE197677, GSE207422, GSE222078, GSE201047, GSE165897. Any other relevant data can be found in the article, supplementary information, or can be requested from the corresponding author.
