# Supplementary material for: Pan-cancer transcriptional atlas of minimal residual disease links DUSP1 to chemotherapy persistence
Source: Exp Hematol Oncol. 2024 Apr 16;13:42. doi: 10.1186/s40164-024-00509-3 (PMC11020193; doi:10.1186/s40164-024-00509-3)
Supplement: Supplementary file 5 — Additional file 5: Figure S1. Canonical pathway signature scores, immune infiltrates and modulators changes induced by chemotherapy. A Heat map illustrating log2(FC) of 10 most widely significantly differentially expressed canonical pathway signature scores across 17 datasets. B Heat map shows the changes in expression (log2(FC)) of inhibitory and stimulatory immune modulators in patients before and after chemotherapy. C Heat map shows the changes in expression (log2(FC)) of seven immunogenic cell death (ICD) modulators between post- and pre-chemotherapy patients. Figure S2. Predictivity of features in pre- and post-chemotherapy samples for drug response and survival. A Distrbution of z score of whole transcriptomic genes for the prediction of overall survival (OS), recurrence free survival (RFS), recurrence (Re) and drug response (DR) in pre-chemotherapy and post-chemotherapy patients samples across nine datasets in three cancers. B Radar plot showing the percentage of significant gene (p<0.05) for the prediction of OS, RFS, Re and DR in pre-chemotherapy and post-chemotherapy patients samples across 9 datasets in three cancers. C Bar plot displaying the fraction of shared significant prognostic genes (overlap) between significant genes derived from patient samples before chemotherapy and significant genes derived from patient samples after chemotherapy. D The number of significant prognostic genes in patient samples before and after chemotherapy was compared across nine datasets in three types of cancer. Each dot represents one dataset. E Kaplan-Meier plots displaying the prognostic ability of ADH in pre-chemotherapy patient sample and in post-chemotherapy patient sample in dataset GSE146965. F Pearson correlation between the gene expression changes (log2(FC) of post- vs pre-chemotherapy) and the z score of genes in COX regression models for OS in pre-chemetherapy patient samples. G Heat map showing the z score of immune infiltrates for prediction of OS, RFS, Re an [file 40164_2024_509_MOESM5_ESM.docx]

**
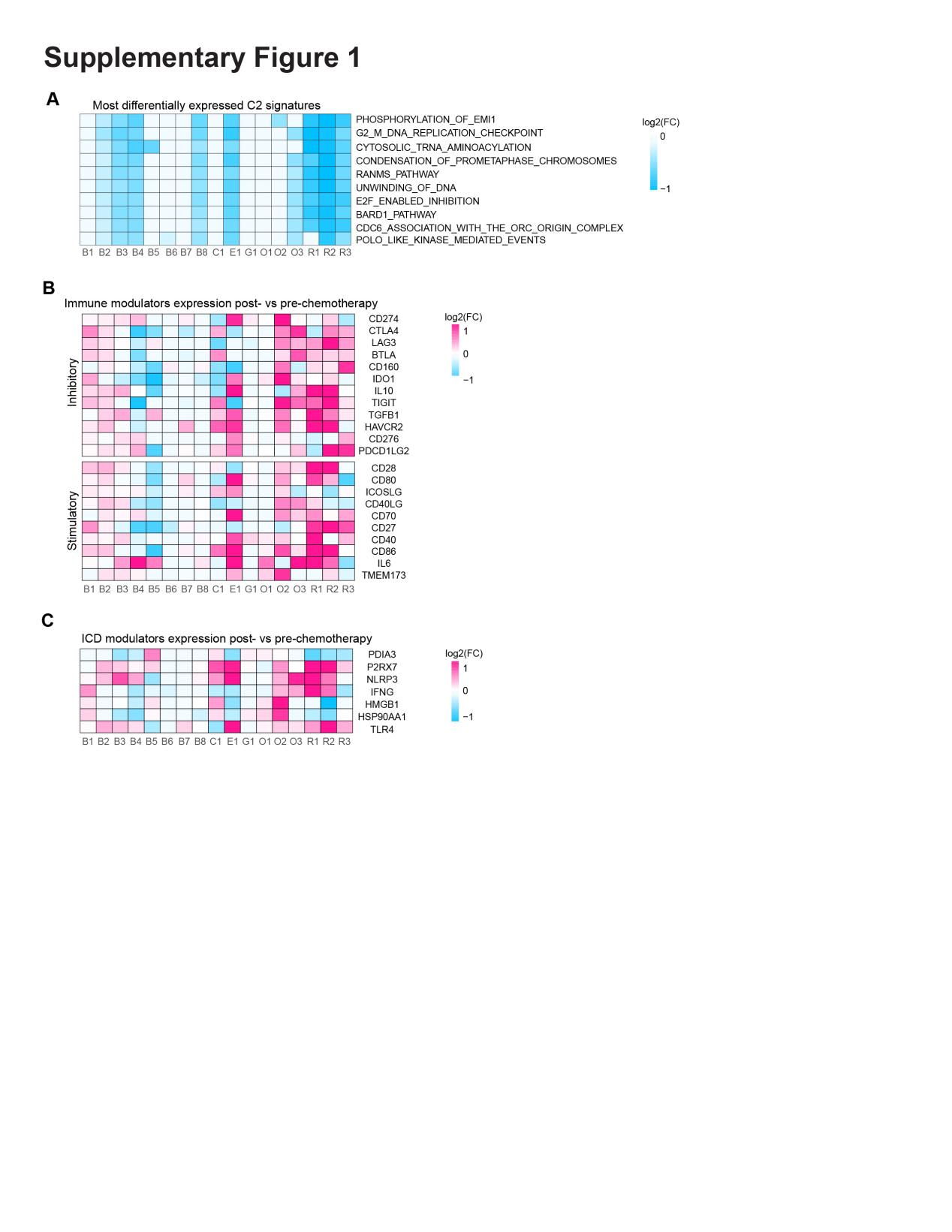
Figure S1. Canonical pathway signature scores, immune infiltrates and modulators changes induced by chemotherapy**

1. Heat map illustrating log2(FC) of 10 most widely significantly differentially expressed canonical pathway signature scores across 17 datasets.
2. Heat map shows the changes in expression (log2(FC)) of inhibitory and stimulatory immune modulators in patients before and after chemotherapy.
3. Heat map shows the changes in expression (log2(FC)) of seven immunogenic cell death (ICD) modulators between post- and pre-chemotherapy patients.


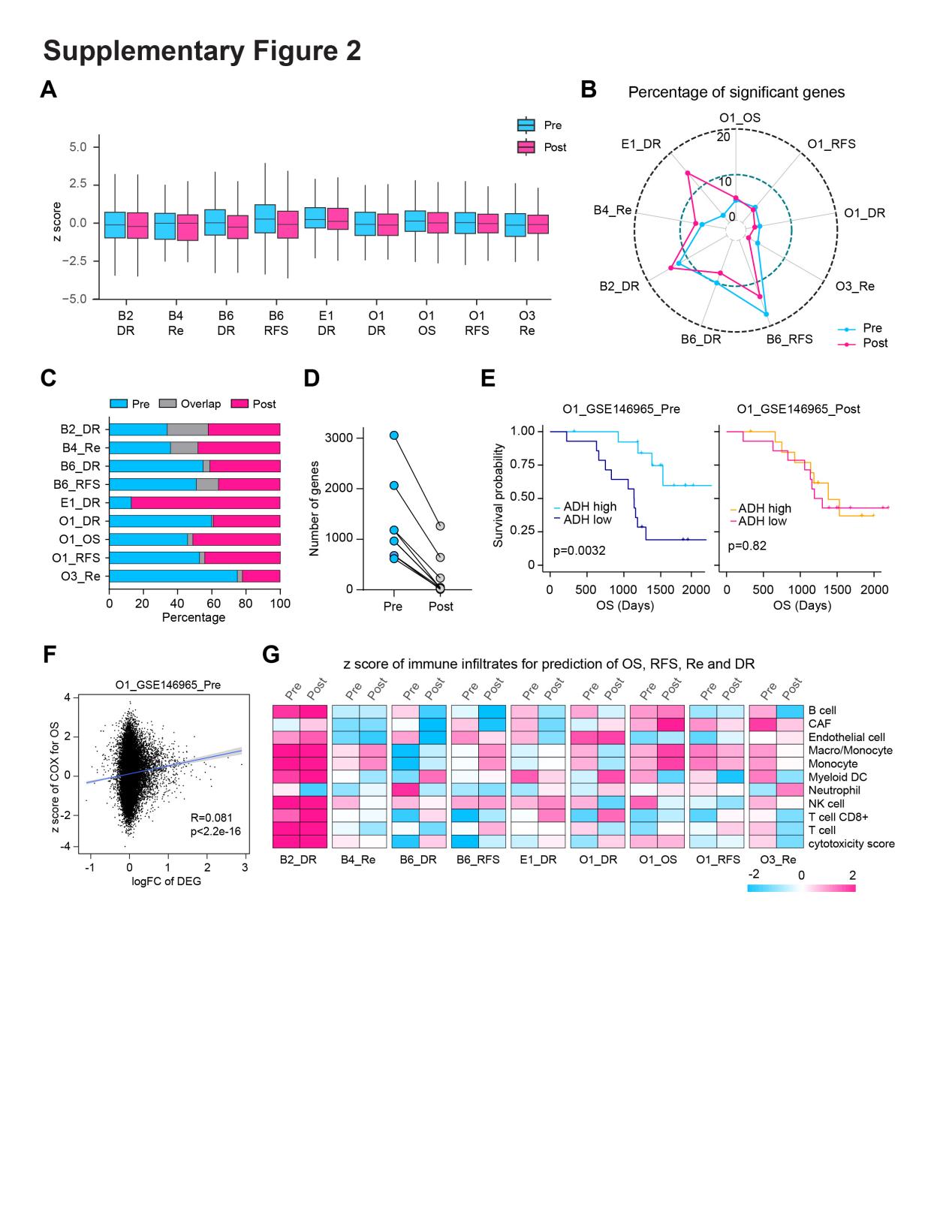


**Figure S2. Predictivity of features in pre- and post-chemotherapy samples for drug response and survival**

1. Distrbution of z score of whole transcriptomic genes for the prediction of overall survival (OS), recurrence free survival (RFS), recurrence (Re) and drug response (DR) in pre-chemotherapy and post-chemotherapy patients samples across nine datasets in three cancers.
2. Radar plot showing the percentage of significant gene (p<0.05) for the prediction of OS, RFS, Re and DR in pre-chemotherapy and post-chemotherapy patients samples across 9 datasets in three cancers.
3. Bar plot displaying the fraction of shared significant prognostic genes (overlap) between significant genes derived from patient samples before chemotherapy and significant genes derived from patient samples after chemotherapy.
4. The number of significant prognostic genes in patient samples before and after chemotherapy was compared across nine datasets in three types of cancer. Each dot represents one dataset.
5. Kaplan-Meier plots displaying the prognostic ability of ADH in pre-chemotherapy patient sample and in post-chemotherapy patient sample in dataset GSE146965.
6. Pearson correlation between the gene expression changes (log2(FC) of post- vs pre-chemotherapy) and the z score of genes in COX regression models for OS in pre-chemetherapy patient samples.
7. Heat map showing the z score of immune infiltrates for prediction of OS, RFS, Re and DR in pre-chemotherapy and post-chemotherapy patients samples across nine datasets in three cancers.
